# Supplementary material for: Locomotion optimizes sensory representations through a computational principle shared by rodents and primates
Source: Sci Adv. 2026 Aug 28;12(35):eaed4172. doi: 10.1126/sciadv.aed4172 (PMC13524049; doi:10.1126/sciadv.aed4172)
Supplement: Supplementary file 1 [file sciadv.aed4172_sm.pdf]

Science Advances
AAAS

Supplementary Materials for

Locomotion optimizes sensory representations through a computational principle shared by rodents and primates

Jonathan M. Gant and Wiktor F. Młynarski

Corresponding author: Wiktor F. Młynarski, mlynarski@bio.lmu.de

Sci. Adv. **12**, eaed4172 (2026)
DOI: 10.1126/sciadv.aed4172

This PDF file includes:

Figs. S1 to S6

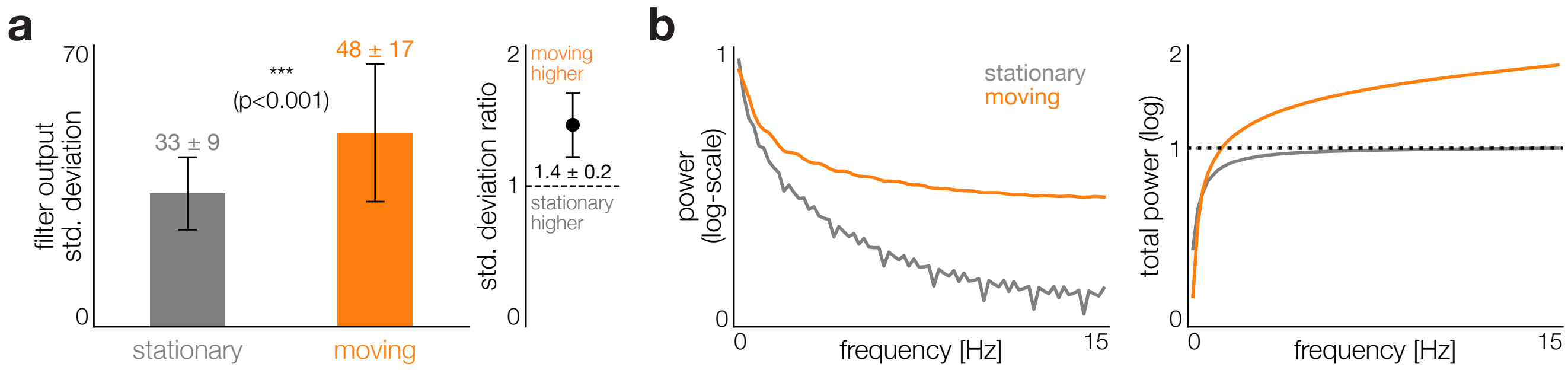

Figure S1: Simulated eye movements do not qualitatively change the impact of locomotion on sensory statistics. We simulated random eye movements that match the distribution of saccade sizes and frequencies observed in mice (*1*). Specifically, the center point of the filter was modified at fixed time intervals (1 second for moving videos and 2 seconds for stationary videos) by adding a randomly signed variable sampled from a Gaussian distribution with a mean of 10 degrees and standard deviation of 3 degrees in the horizontal dimensions and a mean of 1 degree and standard deviation of 0.3 degrees in the vertical dimension. The positions of the filters were clipped such that the filter lay within the bounds of the video frame. **a)** Left panel: Average standard deviation of a large Gabor filterbank where the spatial frequency ranged from 0.03 to 0.12 cpd in stationary (gray) and moving (orange) conditions. The numerical value of the average and the standard deviation of the average are above the bars. The average standard deviation in the moving condition was significantly higher than in the stationary condition (paired t-test, t-statistic=81.27). Right panel: ratio of the average standard deviation of Gabor filter outputs during movement to the average during the stationary condition. The numerical value of the average ratio and the standard deviation of the average ratio are below the point. Error bars indicate standard deviation. **b)** Left panel: the average power spectrum of the outputs of a large Gabor filterbank where the spatial frequency ranged from 0.03 to 0.12 cpd during locomotion (orange) and stationary (gray) conditions. Right panel: the total power (i.e. the integral) of the average spectrum as a function of the frequency for the stationary (gray) and moving conditions (orange).

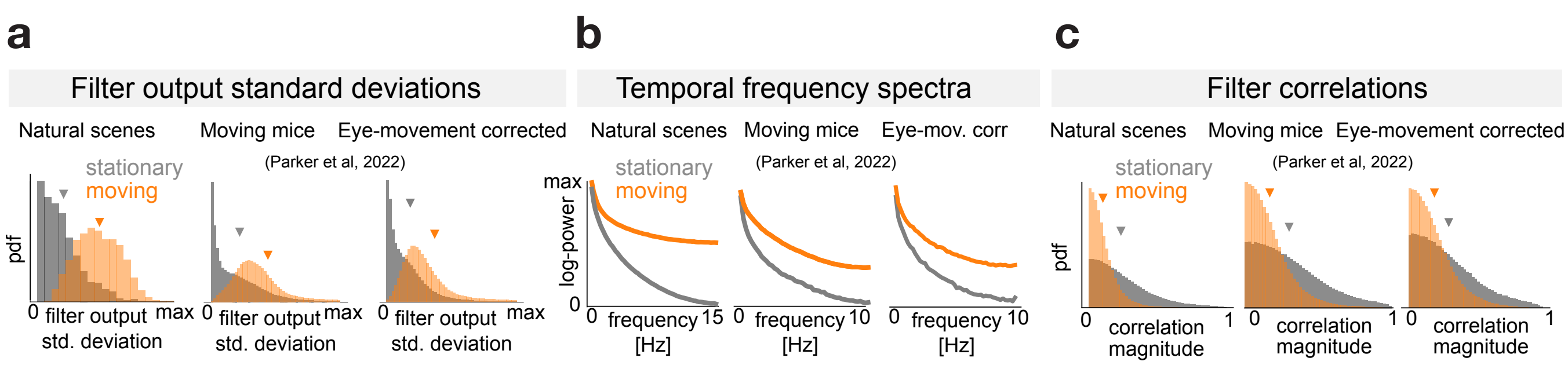

Figure S2: Comparison of distributions of statistical features between natural scene recordings and videos from the perspective of moving mice before and after correction for eye movements (data from (*2*)). To correct for the eye position, for each mouse we trained the "shifter network" that maximizes the predictive strength of a Generalized Linear Model (GLM) of the neural population activity. We used the code and followed the procedure described in (*2*). A range of L2 regularization parameters for the GLM weights ($\lambda \in [0.01, 1000]$) were used and the network with the lowest test loss was used to correct the "world-view" camera videos for eye movements. Using the eye-movement corrected videos, we followed the same procedure as in the analysis of data from freely moving mice (see above). Correcting for eye movements in freely moving mice does not qualitatively change the impact of locomotion on the statistics of sensory inputs. **a)** Standard deviations of filters (triangles denote averages). **b)** Temporal spectra of filter outputs. **c)** Magnitudes of pairwise correlations between filter outputs (triangles denote averages).

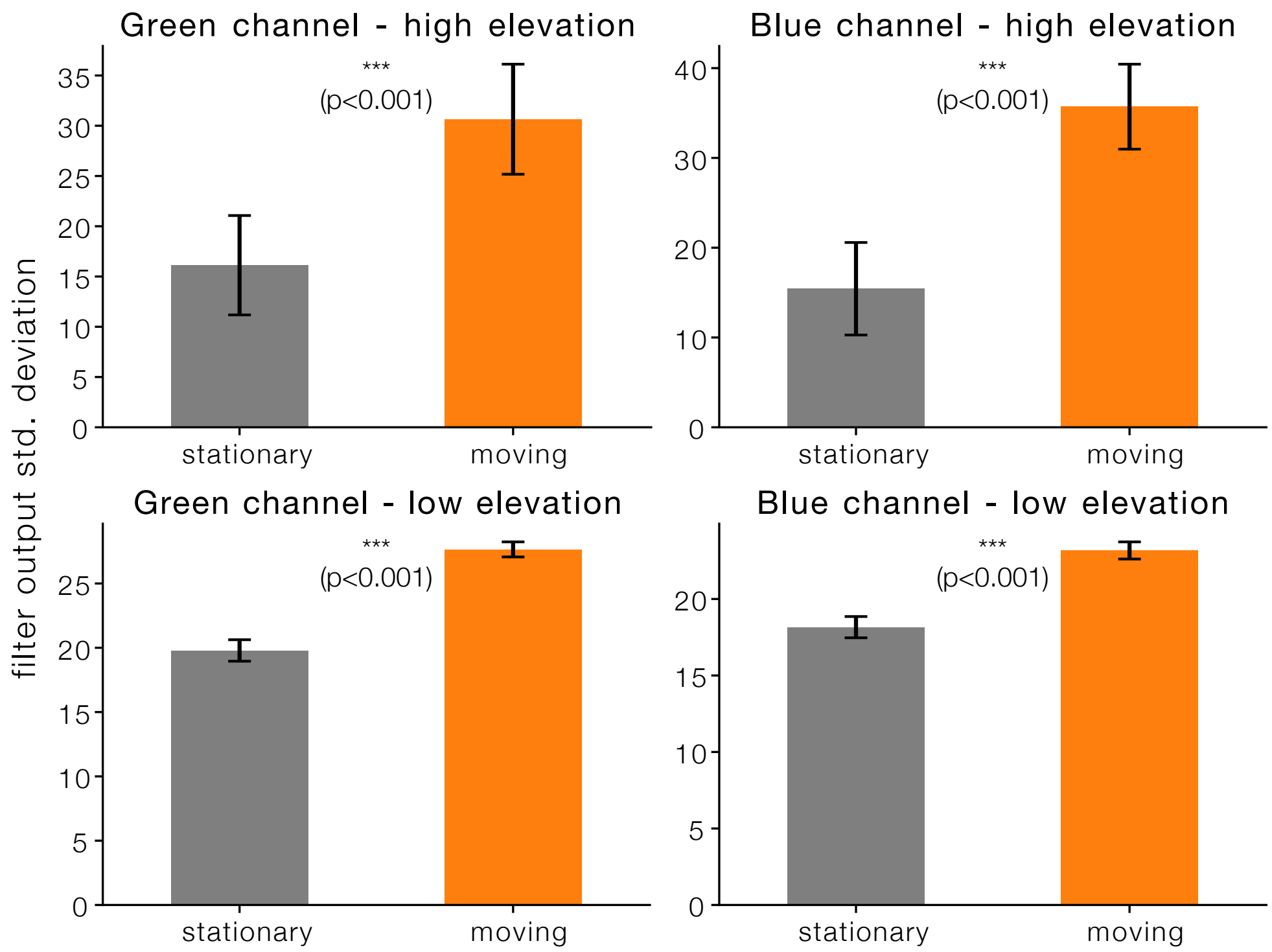

Figure S3: Color vision and elevation do not qualitatively change the impact of locomotion on the standard deviation of filter outputs. The green and blue channels of the natural videos were extracted and the outputs of a bank of Gabor filters were computed for videos recorded in 5 different natural environments in two movement conditions (for a total of 10 videos). The filterbank spanned 8 orientations $\theta \in \{0, 22.5, 45, 67.5, 90, 112.5, 135, 157.5\}$ deg, had a fixed phase of zero, and a spatial frequency of 0.1 cpd. The outputs were computed from two positions in the visual scene: high and low elevation (1/4 and 3/4 of the vertical dimension respectively, centered in the horizontal dimension). Following the same analysis explained in the methods, the filter outputs were segmented into 5 second windows and the standard deviations per window computed. The average standard deviation of filter outputs was computed across windows and environments for each movement condition, color channel, orientation and position and are summarized by the bar plots which are organized by color channel and position and report the mean of the average standard deviations across filter orientations. The columns show the results for the green channel (left) and blue channel (right). The rows show the results for the high elevation (top) and low elevation (bottom). For all color channels and elevations, the filter output standard deviations are significantly higher while moving than while stationary (paired t-test, t-statistic: 33.854 (upper left), 53.256 (upper right), 19.006 (lower left), 17.113 (lower right)).

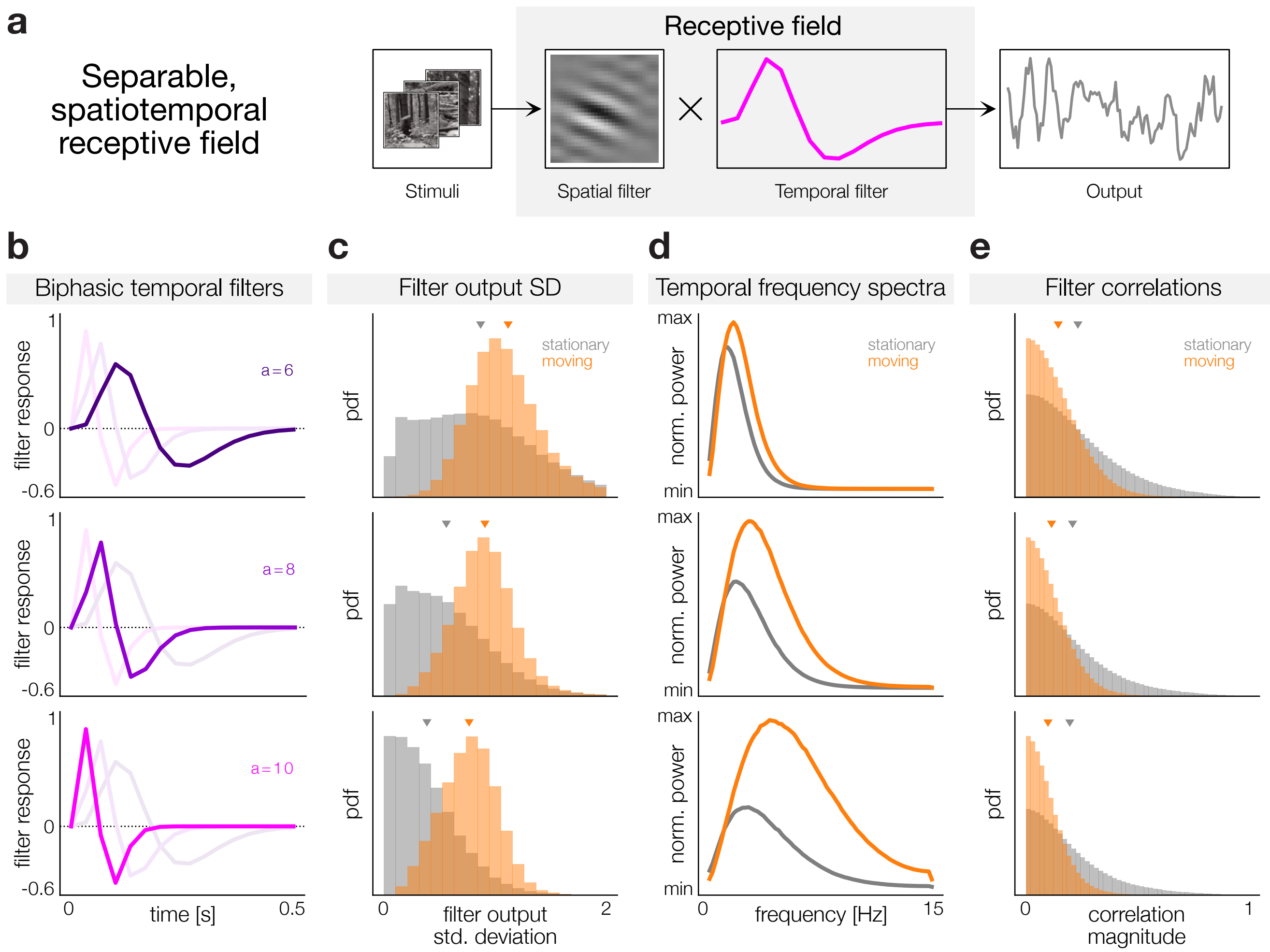

Figure S4: Outputs of spatiotemporal filters are systematically influenced by of locomotion. The outputs of the spatial ICA filters were temporally filtered with biphasic temporal filters reminiscent of temporal receptive fields. The biphasic temporal filters are parameterized as $F(t) = \exp(-at)\left(\frac{(at)^5}{5!} - \frac{(at)^7}{7!}\right)$, where the parameter a controls the timescale of the filter as in (*3*). We used temporal filters with a length of 0.5 seconds and three values of $a \in \{6, 8, 10\}$ to match the timescales of temporal receptive fields in mice. The temporally filtered outputs were then processed in the same manner described earlier for the analysis of the Gabor filterbank outputs and ICA filters to compute the filter output standard deviations, temporal spectra, and filter correlations. **a)** Schematic showing the application of a separable, spatiotemporal receptive field to video stimuli. **b)** Biphasic temporal filters with three different timescales. **c)** Standard deviations of temporally filtered ICA filter outputs (triangles denote averages). For all values of a the average filter output standard deviation is higher during locomotion than in the stationary condition consistent with the previous results (Fig. **??**). **d)** Temporal spectra of temporally filtered ICA filter outputs. **e)** Magnitudes of pairwise correlations between temporally filtered ICA filter outputs (triangles denote averages).

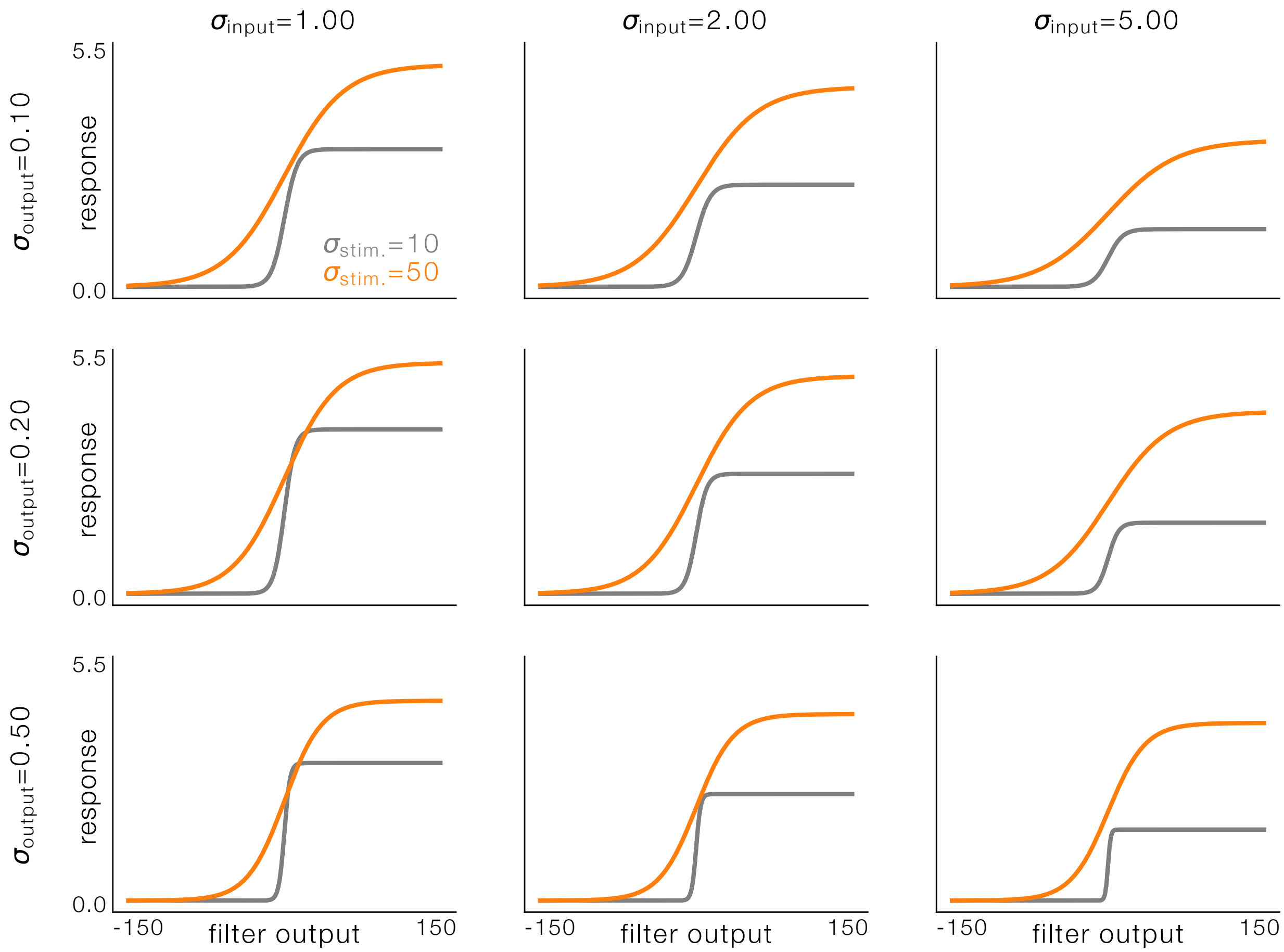

Figure S5: Noise does not qualitatively impact the gain increase of optimal nonlinearities during locomotion. Optimal nonlinearities were computed for low and high variance stimuli for a range of input and output noise levels. For both the input and output noise Gaussian random variables were used. The stimulus standard deviations of $\sigma_{\text{stim.}} \in \{10, 50\}$ were chosen to roughly approximate typical values of filter output standard deviations to stationary and moving videos respectively. The standard deviations of the input noise used were $\sigma_{\text{input}} \in \{1, 2, 5\}$ and the output noise standard deviations used were $\sigma_{\text{output}} \in \{0.05, 0.1, 0.2\}$. For the input signal, 10^6 samples were randomly chosen from a Gaussian distribution. A grid search was performed over the nonlinearity parameters with 100 logarithmically spaced k values from 0.01 to 1 and 100 linearly spaced L values from 0.1 to 10. The mutual information was estimated directly from the discretized joint and marginal distributions of the stimuli and firing rates with a fixed binning scheme with $2,000$ bins ranging from -150 to 150 for the stimuli and $2,000$ bins ranging from 0 to 10 for the firing rates. The average firing rate and utility were computed in the same manner as before with $\lambda_r = 0.5$. With increasing input noise the maximal firing rate of the nonlinearities tends to decrease so as not to amplify the input noise. With increasing output noise, the slope of the nonlinearities tends to increase, that is to say they get steeper.

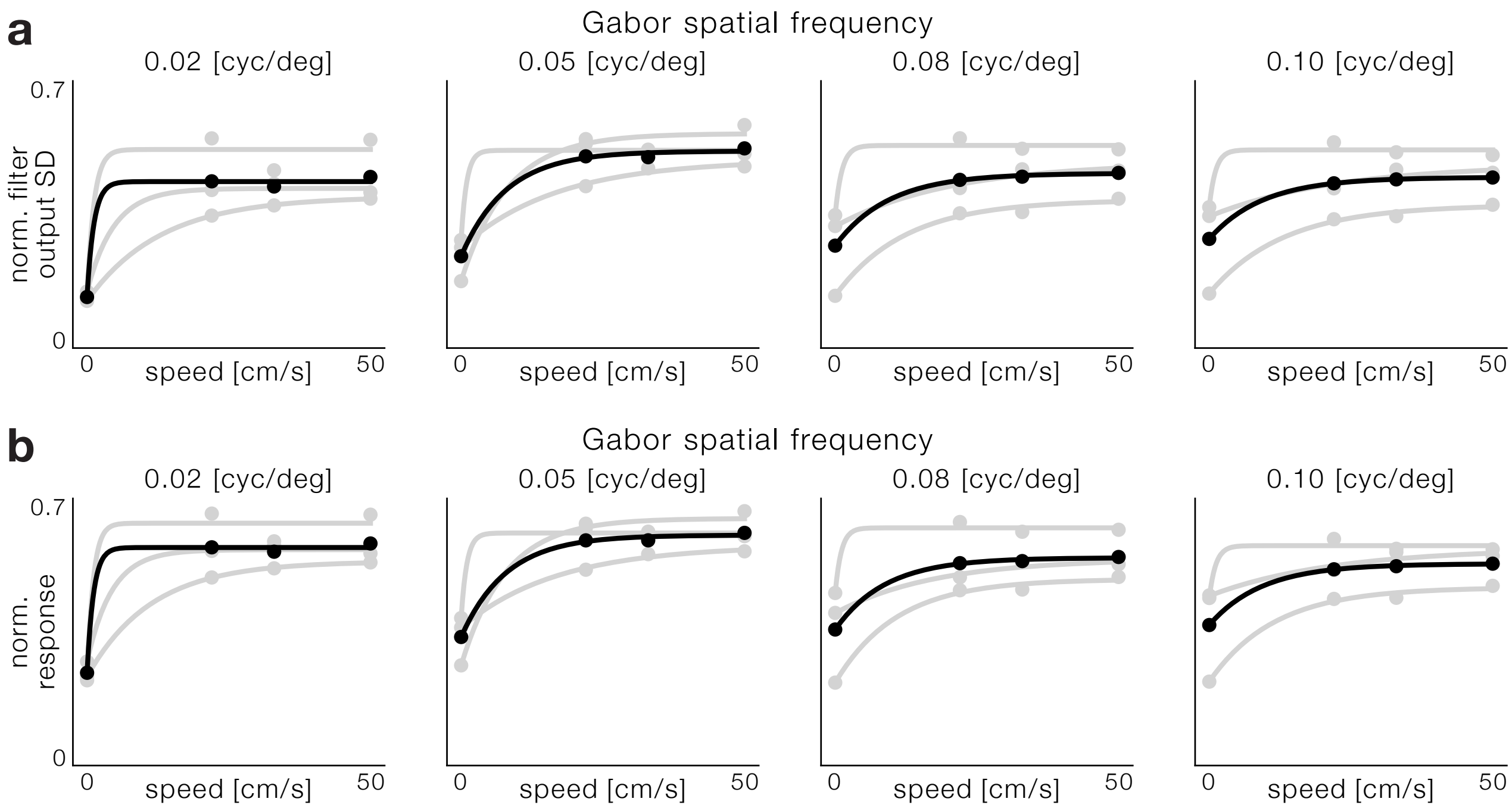

Figure S6: Locomotion increases firing rates at the onset of locomotion across different spatial frequencies and environments. The same procedure as in the methods section was followed for videos taken at 3 different speeds in three different environments: forest, field, and orchard. Rather than taking the average of the standard deviation of filter outputs across environments, the average standard deviation of filter outputs was computed for each environment separately. The average standard deviation as a function of movement speed was then fit with an exponential function as before for each environment. For comparison the mean over the average standard deviations per environment and speed were also computed and fit with an exponential function. The optimal nonlinearities were chosen and the firing rates were computed in a similar manner as before, but per environment rather than over all environments. Lastly the mean over the average firing rates per environment was computed and fit with an exponential as well. **a)** Average standard deviations of a bank of Gabor filters as a function of running speed for different spatial frequencies. Gray lines/points are for individual environments and the black line/points are averaged over environments. **b)** Average normalized response of the optimal nonlinearities for a bank of Gabor filters as a function of running speed for different spatial frequencies. Gray lines/points are for individual environments and the black line/points are averaged over environments. For this analysis $\lambda_r = 1$.
